# Hydration–Dehydration Dynamics in the Desiccation-Tolerant Moss *Hedwigia ciliata*

**DOI:** 10.3390/plants14243849

**Published:** 2025-12-17

**Authors:** Pragya Singh, Djordje P. Božović, Deepti Routray, Michal Goga, Martin Bačkor, Marko S. Sabovljević

**Affiliations:** 1 Department of Plant Biology, Institute of Biology and Ecology, Faculty of Science, Pavol Jozef Šafárik University, Mánesova 23, 041 67 Košice, Slovakia; pragya.singh@student.upjs.sk (P.S.); deepti.routray@upjs.sk (D.R.); michal.goga@upjs.sk (M.G.);; 2 Institute for Botany and Botanical Garden, Faculty of Biology, University of Belgrade, Takovska 43, 11000 Belgrade, Serbia; djordje.bozovic@bio.bg.ac.rs; 3 Center for Interdisciplinary Biosciences, Technology and Innovation Park, Pavol Jozef Šafárik University, Jesenná 5, 041 54 Košice, Slovakia; 4 Institute of Biotechnology, Faculty of Biotechnology and Food Science, Slovak University of Agriculture, Tr. A. Hlinku 2, 949 76 Nitra, Slovakia; 5 Center of Plant Biotechnology and Conservation (CPBC), Takovska 43, 11000 Belgrade, Serbia

**Keywords:** bryophytes, stress, anabiosis, photosynthesis, water content

## Abstract

The desiccation tolerant moss *Hedwigia ciliata* was investigated to assess its early reaction to water stress in controlled conditions. Thus, various photosynthetic parameters and water contents were studied during a selected time frame within 48 h of two main events, namely water loss after full hydration and activity (preparation for anabiosis) and water uptake after the state of anabiosis. The observations of the changes in the photosynthetic efficiency of *Hedwigia ciliata* in response to fluctuating relative water contents (RWCs) during rehydration and dehydration periods provide valuable insights into the species’ physiological adaptation mechanisms. *Hedwigia ciliata* rapidly uptakes water upon anabiosis, and despite being poikilohydric, it is able to retain water for a prolonged period compared to the water availability in its immediate environment. The delayed decrease in relative water content (RWC) corresponds to the photosynthetic parameters and preparation for anabiosis, including the maintenance of photosystems and general cell integrity. The low values of non-photochemical quenching during desiccation imply limited photoinhibition and effective photoprotective regulation. Conversely, the rather high values of the fluorescence decline ratio during rehydration reflect the efficient recovery of photosynthetic performance. The postponed physiological shutdown while drying and photosynthetic activity in the early dehydration phase suggest anticipatory acclimation to desiccation, i.e., non-active phase anabiosis. *Hedwigia ciliata* is a desiccation-tolerant moss, with anatomical, morphological, and eco-physiological adaptations, making it a useful model species in drought and abiotic stress studies of bryophytes in a rapidly changing environment.

## 1. Introduction

Water plays a crucial role in the proper functioning of photosystems and other significant physiological processes in plants. Hence, the absence or insufficient availability of water has a major impact on plant vitality [1]. Climate change, including water shortages, is known as one of the most critical threats to nature and the survival of diversity [2,3]. Water scarcity, otherwise known as drought, is a key indicator of recent and rapid climate change, which severely affects numerous plant life forms [4]. Over the past few decades, drought stress has increased significantly, with a 29% rise in frequency since 2000 according to a UN report [5]. As a result, plant populations face severe consequences of such intensified drought conditions in their natural habitats, including disappearance, exposure to sub-lethal conditions, survival struggle, or migration to other places if ecologically available [6]. Furthermore, the resulting impacts are not only limited to natural plant communities, but also to crop species [3]. For example, a single week of drought stress in major crop-producing areas may reduce yields by approximately 8–10%, a loss which is predicted to increase by 50% in the near future [7].

Bryophytes are an ancient and rather successful group of terrestrial plants which have colonized almost all habitats. They also successfully cohabit in a wide range of extreme climatic conditions, including cold and dry environments [8]. As poikilohydric plants, bryophytes are generally small and green, and are classified into three major lineages: liverworts, mosses, and hornworts [9]. The effects of drought stress on bryophytes, including their survival as populations, species, and vegetation components, remain poorly understood, particularly regarding their morphological and physiological adaptations [10,11,12]. In recent years, the efficiency and brilliance of these tiny plants have gathered increasing scientific attention in different fields of research. Mosses form an essential part of many ecosystems due of their significant contribution to soil erosion prevention, nutrient cycling, and carbon balance, as well as other key processes [13,14,15]. They are non-vascular plants, and although the presence of hydroids and leptoids suggest some type of internal water and nutrient transport, they lack a fully developed vascular system for the supply of water [16]. They principally absorb water through their thalli (i.e., body), and thus, strongly rely on external humidity [16]. The total water content in bryophytes is categorized into two components, i.e., external and internal. In mosses, the distribution of water is external (ectohydric) [17,18]. The external water-holding capacity within the bryophyte species varies and mainly depends on the growth pattern, its density, and the type of branching [19]. The absence of a cuticle and the higher ratio of surface-to-volume cause quick drying out and water-deficit conditions [20]. However, many moss species can lose maximum cellular water without dying and resume normal photosynthesis and metabolism upon rehydration (e.g., [21]). Thus, hydration of the moss body is closely related to the humidity in the nearby environment, a phenomenon known as poikilohydry [9]. During dehydration, moss organisms enter a state named anabiosis, where they appear lifeless [9]. However, upon rapid rehydration, moss organisms recover and quickly recuperate to fully functional and living organisms [22]. Within the bryophytes, mosses seem to be an especially drought stress-tolerant species. The anabiosis state allows them to endure such unfavorable conditions by halting metabolic activities and conserving energy until the environment becomes favorable, so as to minimize any possible damage from drying out [9]. It is essential for plants to protect their vital structures, such as cell membranes, and severe dehydration can lead to disruption of the lipid bilayer structure. The prevention of compromising membrane integrity and altering their fluidity and function is of vital importance [23,24]. In a fully hydrated state, all mosses have a relative water content (RWC) close to 100%, but it gradually decreases, often dropping to below 50–60% within a few hours of desiccation [6,25]. The response to desiccation has been studied in some bryophytes, such as moss species *Bryum argenteum* Hedw., *Barbula unguiculata* Hedw., and *Erythrodontium julaceum* (Schwaegr.) Par., showing that the RWC declines to approx. 40–45% after 72 h of stress exposure [26]. A comprehensive overview of this topic and the species studied can be found in the study by Proctor et al. [27].

In addition, studies of mosses from the genus *Syntrichia* have revealed desiccation resistance in the tested species [28]. Water content studies and analyses of the water relations and photosynthetic characteristics of bryophytes during desiccation and rehydration provide deeper insights into their physiological adaptations and desiccation coping mechanisms [19,29]. Photosynthetic activity typically declines beyond a certain threshold of water loss. Bryophyte species which can conserve their photosynthetic apparatus during desiccation have been categorized as homoiochlorophyllous [30].

*Physcomitrium patens* (Hedw.) Mitt., (syn. *Physcomitrella patens* (Hedw.) Bruch and Schimp.), a model species studied in relation to environmental stress, showed sensitivity and tolerance to desiccation, as depicted by the water content variation, ion leakage, and cellular damage during rehydration and dehydration [31].

The acrocarpous moss *Hedwigia ciliata* (Hedw.) P.Beauv. selected for this study belongs to the Hedwigiaceae family. This is an epilithic moss, which grows on acidic rocks under direct sunlight with a life cycle span of several years. It can support full sun conditions, but also some shade. It has been reported in ecologically diverse conditions and it is able to survive in both highly moist and very dry conditions, but generally avoids permanently humid sites (e.g., [32,33]). This moss remains persistent even under thick layers of snow and freezing conditions during winter by entering an anabiotic state and then resuming normal functions upon the return of favorable conditions. Several morphological features, such as imbricate leaves, cell papillosity, or exerted leaf tips, are considered to play a role in water collection during wet periods, and water conservation and supply during dry intervals. Hyaline leaf tips and grayish coloration, when dry, additionally aid survival in harsh and changeable environments. The loose grayish-green large plants of *H. ciliata* often grow in patches of curved stems irregularly branched from erect to pendant. These intermingled shoots form tufts which serve to additionally down-regulate water loss and evaporation in this poikilohydrous moss species.

Water balancing during hydration/rehydration processes related to photosynthetic parameters is rather insufficiently documented in mosses. The aim of this work is to examine the biological features and survival strategy of the desiccation-tolerant moss *H. ciliata* both prior to and immediately after anabiosis. Thus, we studied its relative water content and photosynthetic rate during the initial (early) phases of rehydration and dehydration (48 h). The photosynthetic rate was inferred through the dynamics of various parameters such as maximum quantum yield of photosystem II (QY max), non-photochemical quenching (NPQ), photochemical quenching (qP), and the fluorescence decline ratio (Rfd). The comparatively studied early water loss/uptake effects on photosynthesis in bryophytes are not often focused on mosses and, to our knowledge, this is the first time they are investigated on a moss model species, namely, *H. ciliata*.

## 2. Results

### 2.1. Relative Water Content

**Figure 1:**
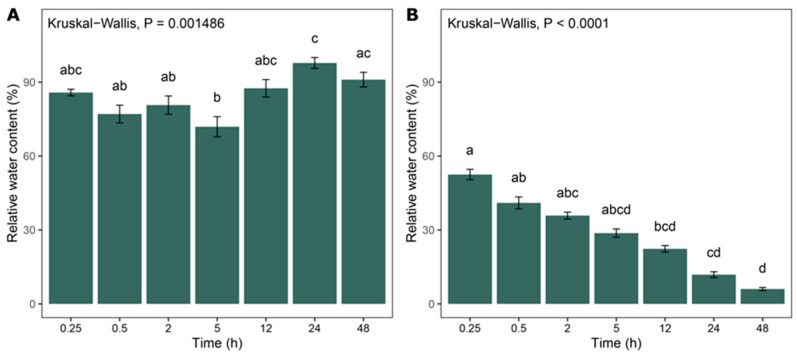
The relative water content of *H. ciliata* during rehydration (**A**) and dehydration (**B**) over time. The data are presented as mean ± standard error (SE). Different letters above the bars indicate statistically significant differences between the time points based on Dunn’s post hoc test (*p <* 0.05).

During rehydration (Figure 1A), the relative water content (RWC) did not differ significantly over time. This indicates that the plants successfully rehydrated within just 15 min and reached a sufficiently high RWC (close to 90%). The relative water content in the tested plants did not increase further with prolonged water exposure. In contrast, during dehydration (Figure 1B), a clear time-dependent decline in RWC was observed. The relative water content decreased progressively with increased exposure to air in the absence of water, as expected.

### 2.2. Chlorophyll Fluorescence

**Figure 2:**
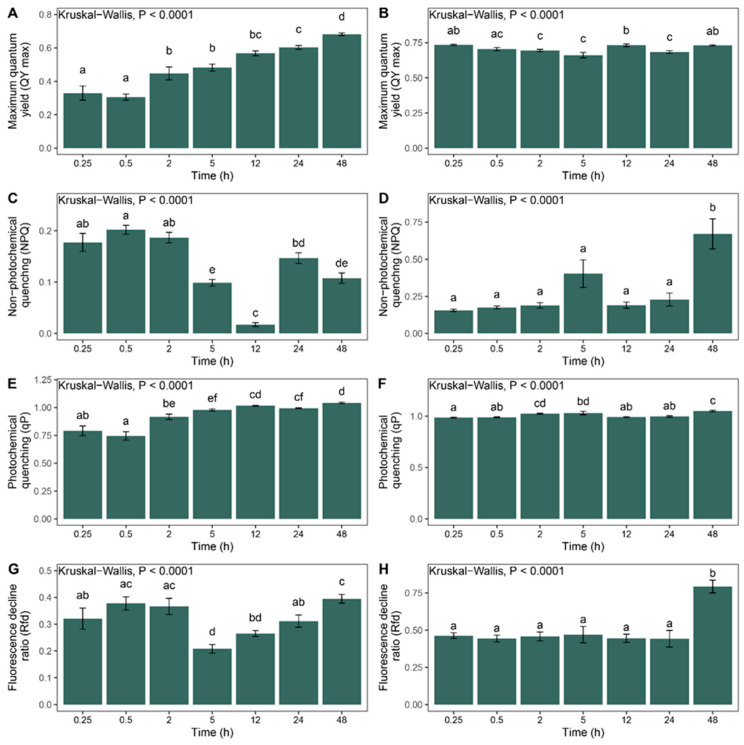
The chlorophyll fluorescence parameters of dark-adapted *H. ciliata* samples during rehydration (**A**,**C**,**E**,**G**) and dehydration (**B**,**D**,**F**,**H**) over time. The maximum quantum yield of photosystem II (**A**,**B**); non-photochemical quenching (**C**,**D**); photochemical quenching (**E**,**F**); and the fluorescence decline ratio (**G**,**H**). The data are presented as mean ± standard error (SE). Different letters above the bars indicate statistically significant differences between the time points based on Dunn’s post hoc test (*p <* 0.05).

The maximum quantum yield of photosystem II (QY max) increased over time during rehydration (Figure 2A), reaching its highest value at 48 h of water exposure. The parameter (QY max) usually refers to the efficiency of photosynthesis, i.e., how effectively absorbed light is converted further for photochemical reactions. This value was significantly higher (*p <* 0.05) than in all the other treatment groups. These results suggest that, although the RWC reaches sufficient levels within just 15 min of rehydration, the recovery and activation of the photosynthetic machinery require more time to fully respond to the hydration event. During dehydration, the maximum quantum yield of photosystem II remained relatively stable (Figure 2B). Even after 48 h without water, the values did not differ significantly from those observed at 15 min, indicating an effective water-retention capacity and desiccation tolerance in the tested species. The ability of plants to prevent damage to photosystem II and to decrease the production of reactive oxygen species (ROS) by the dissipation of received light energy as heat is measured by the non-photochemical quenching (NPQ) parameter. Non-photochemical quenching (NPQ) showed relatively low values (<0.2) during rehydration (Figure 2C), indicating minimal energy dissipation as heat. Similar NPQ levels were observed during dehydration (Figure 2D). An exception was observed in the 48 h group, which exhibited a significant increase (*p <* 0.05) compared to all the other time points. This spike suggests that after prolonged desiccation (48 h without water), the photosynthetic machinery becomes increasingly impaired, and the plant dissipates excess excitation energy to prevent photo-oxidative damage. Another photosynthesis parameter, photochemical quenching (qP), shows how much of the received light energy is used in photochemical reactions by plants, since part of it can be lost as heat or fluorescence. Photochemical quenching (qP) showed a slight increase over time during rehydration (Figure 2E), indicating the gradual restoration of the open PSII reaction centers and improved photochemical efficiency as the photosynthetic apparatus recovers. During dehydration (Figure 2F), the qP values remained relatively stable, although a small but significant increase was observed after 48 h compared to the 15 min group (*p <* 0.05). The fluorescence decline ratio (Rfd), a value indicating plant (photosystem) vitality, fluctuated slightly during rehydration (Figure 2G). During dehydration (Figure 2H), the Rfd values remained relatively stable across the time points, except at 48 h, where a significant increase was observed (*p <* 0.05), mirroring the trend seen in NPQ. Although high Rfd values are generally associated with good plant fitness and lower stress, in this case, a decrease in the steady-state fluorescence level (Ft) due to stress-induced factors such as enhanced non-photochemical quenching can cause the Rfd to rise. Therefore, the elevated Rfd values at 48 h of dehydration likely result from a significant reduction in Ft rather than improved photosynthetic performance, indicating increased stress and the compromised functioning of the photosynthetic apparatus.

## 3. Discussion

The degree of moisture in moss plants’ immediate surroundings causes changes in the hydration levels in many species. Consequently, bryophytes frequently encounter drying circumstances and regularly undergo desiccation. Moss species are especially susceptible to variations in weather and habitat conditions because of their passive reliance on external humidity. One of the most common techniques for determining a plant’s water-holding capacity is to measure its relative water content [34]. Our study focuses on the relation between RWC and photosynthesis efficiency changes during the transition into and out of anabiosis.

The data achieved by measuring the RWC in *H. ciliata* during the rehydration experiment in our study show a higher water absorption and retention capacity over a short period of water exposure, which is maintained for a longer period of time in a humid environment. The presence of whitish leaf tips and hyaline cells in *H. ciliata* contributes to the immediate rise in the overall hydration status of the moss body by catching the morning dew and dissipating the direct light. Thus, the increase in the RWC occurs within a few minutes [35]. Hyaline cells account for up to approx. 10% of the total water-holding capacity and facilitate passive water exchange through the pores [36,37]. In some species of the genus *Syntrichia*, i.e., *S. papillosissima* (Copp.) Loeske, *S. bartramii* (Steere) R. H. Zander and *S. princeps* (De Not.) Mitt., the hyaline basal cells have been suggested to play a major role in the rapid absorption of water in short durations, hence, the elevated RWC [19]. On the other hand, during the dehydration experiment, with the passage of time, the RWC decreased gradually in *H. ciliata* as the plants lost water and dried out at room temperature. The rate of drying is strongly influenced by the ambient relative humidity; under conditions that favor moisture loss, the specimen rapidly attains an equilibrium with the water potential of the surrounding air. In addition, the RWC is primarily driven by the distinct morphological, anatomical, and physiological characteristics of mosses, specifically in desert habitats [38,39]. Moss species from arid or extremely dry regions, for example, *Bryum argenteum*, *Barbula unguiculata*, and *Erythrodontium julaceum*, have also been studied in the past to assess their RWC during a hydrated state and a desiccation stress period of up to several hours. However, in these three moss species, the RWC rapidly declined with the increase in the drying period. During the rehydration period, these species showed a high RWC with a short exposure to water and no remarkable differences were observed with further absorption of water up to several hours [26], similar to the moss in our study. Moss species like *Tortella squarrosa* (Brid.) Limpr., *Tortella tortuosa* (Hedw.) Limpr. and *Campylopus pyriformis* (Schultz) Brid., from dry regions, have a denser cushion or mat-like gametophyte structure. This is not a growth pattern characteristic of *H. ciliata*, suggesting rather different survival strategies in water retention and desiccation tolerance. Thicker growth forms assist in maintaining internal microclimatic stability under uniform desiccation conditions, regulating humidity, temperature, and airflow, which, in turn, modulates the physiological and ecological responses to water-deficit conditions [39]. It remains unclear as to whether moss species with looser growth forms are able to absorb water and water spray as quickly as those with thicker mats. During the measurement of RWC in this study, we observed the slower drying rate and higher water retention capacity of *H. ciliata* despite its loose habitus form. This slow phenomenon might serve as a preparatory mechanism for anabiosis, functioning as an adaptive/protective strategy to minimize cellular damage, similar to the post-rehydration phase following prolonged desiccation during summer. Slow entry into anabiosis can be crucial for survival, since all the systems gain sufficient time to be well prepared for inactivity.

Chlorophyll fluorescence in *H. ciliata* demonstrates its photosynthetic responses and ability to sustain during slow desiccation while transitioning to anabiosis. As the moss body started absorbing sufficient water from the onset of rehydration, *H. ciliata* showed a progressively higher photosynthetic output over time. Photosynthetic efficiency, typically measured by quantum yield (QYmax), reached its maximum point with respect to the hydration period. Interestingly, no significant impacts of drying out on QYmax were documented during the desiccation period, inferring no harm to PSII and the stability of its photosynthetic apparatus. The shutdown of photosynthesis in bryophytes is a reversible response commonly triggered by dry conditions. Several studies on *Syntrichia* spp. have claimed that the longer the duration of stress, the slower the rate of recovery is, resulting in greater damage, especially to the ultrastructure of the cell membranes and photosynthetic apparatus [40]. Sometimes, plants may act differently and remain in a hyperactive state in order to work more efficiently with the minimum available resources before drying out completely or entering anabiosis [41]. In a similar experiment carried out with a group of six different moss species, it was reported that the rate of recovery after hydration varied among the species. Among the six species, four bryophytes, *Didymodon fallax* (Hedw.) R. H. Zander, *Grimmia lisae* De Not., *Tortella squarrosa*, and *Targionia hypophylla* L., exhibited better and greater recovery of PSII upon hydration after desiccation treatment compared to the other two species, *Hypnum cupressiforme* Hedw. and *Tortella nitida* (Lindb.) Broth. The obtained values in *H. cupressiforme* and *T. nitida* were much lower than the reference range for the evaluation of photosynthetic fitness, with very high NPQ values as a result of the activation of photo-protective mechanisms [42]. These two species are assumed to be able to endure fewer dehydration/rehydration cycles than the other four species.

The lower the values of NPQ are, the higher the resilience of the specific species in the anhydrobiotic state [43,44]. Conversely, in *H. ciliata*, the NPQ values recorded in the rehydrated periods imply that the defensive strategies remain active while some water is still present in the tissues. As the water potential drops below −15 MPa, most cellular processes come to a halt. Therefore, these processes are essential to protect the moss from desiccation and must be prepared before the critical threshold is reached [43,44]. A higher photochemical quenching coefficient was observed in *H. ciliata* during both the rehydration and gradual desiccation periods, indicating the better performance of the PSII centers. This means that the changing conditions, i.e., from anabiosis to rapid hydration followed by very low RWC, did not show any negative impact on the electron transport chain and the use of the absorbed energy for photochemical reactions. At the same time, this also suggests that a slow onset of stress may allow such protective mechanisms/acquired adaptation to prevent any damage to the photosystem. PSII remains active and functional, delaying damage. In a comparative study, moss *Syntrichia ruralis* (Hedw.) F. Weber and D. Mohr showed significant variations in the above variables, indicating the photosystem’s fitness [45]. The results showed different levels of stress experienced by the moss, which to some extent varied depending on the duration of recovery. The same authors suggested that even during hot and dry days when the RWC of the moss body was very low, they remained photosynthetically active [45]. Previous studies on another moss, *Atrichum androgynum* (Müll. Hal.) A. Jaeger, showed an increase in NPQ during rehydrating periods, focusing more on photoprotection and resulting in greater tolerance [46]. The Antarctic moss *Chorisodontium aciphyllum* Broth. demonstrated a similar trend to *H. ciliata,* as NPQ remained constant while the RWC% decreased to minimum levels during desiccation treatment. These findings suggest that the activation of NPQ seems to be an effective stage in photo-protective mechanisms [47].

Like other fluorescence parameters, the fluorescence decline ratio (Rfd) provides insights into photosynthetic performance, particularly in challenging conditions like low RWC and rapid rehydration in bryophytes [48]. The higher Rfd values during rehydration from the onset to several hours later recorded in our study typically demonstrate greater photosynthetic efficiency and healthier photosystems. Higher Rfd values compared to NPQ during the treatment explain the greater efficiency of photosynthetic energy conversion and less energy being dissipated as heat. This may indicate that *H. ciliata* did not suffer extreme photodamage but remained prepared for its mitigation throughout the desiccation period.

Previous studies have reported on the relationship between desiccation resistance and water content in *H. ciliata* along with other moss species from xeric, mesic, and hydric habitats [49,50]. These comparative studies include the changes observed in their physiology and photosynthetic characteristics during desiccation and rehydration. The trend observed in the bryophytes from the xeric habitats was one of being well adapted to dehydration periods unlike the species from the other habitats included in the study. Similarly, *H. ciliata* showed a tolerance of up to a few hours of dehydration and remained metabolically active in the early stages of desiccation while safeguarding its photosystem [49]. This might be explained by the presence of constitutive desiccation-tolerance mechanisms, where protective strategies and structural adaptations help in maintaining cell integrity during dehydration regardless of the environmental conditions [51].

One of the key plant growth regulators, namely, abscisic acid (ABA), enables survival during desiccation in many moss species studied to date [52]. Its role is to upregulate the stress-responsive genes involved in the activation of antioxidant enzymes (SOD, CAT, or APX) and proteins such as LEA and HSPs, boosting the accumulation of protective molecules like sugars, polyphenols, flavonoids, or proline. All these molecules participate in maintaining structure and protecting cellular components during water deficits. ABA also assists in maintaining membrane integrity during shrinkage caused by water loss and in the accumulation of protective lipids to reduce leakage upon rehydration. In mosses, ABA has been shown to be crucial both for inducible desiccation tolerance and the enhancement of constitutive tolerance [53]. It is worth mentioning that ABA also plays a pivotal role upon rewetting, in faster DNA repair, membrane recovery mechanisms, more efficient resumption of photosynthesis, and the reduction in oxidative stress.

Therefore, species with constitutive tolerance like *H. ciliata* are better prepared to survive immediate and severe desiccation, offering them a significant survival advantage in harsh and unpredictable environments [54,55].

The results obtained clearly show that *H. ciliata* has strong desiccation tolerance and maintains photosynthetic efficiency during dehydration and rehydration, thanks to its high RWC, i.e., water retention, active photoprotection (NPQ), and stable PSII performance. Its rapid water uptake and slow drying rate suggest structural and physiological adaptations for survival in dry habitats. However, continued research into the physiological processes and biochemical adaptations of *H. ciliata* could enhance our understanding of various other factors contributing to its tolerance to desiccation.

The findings for *H. ciliata* partly overlap with similar studies on other bryophyte species, such as *Syntrichia caninervis* (Mitt.) Broth., *S. pagorom* (Milde) J.J. Amann, *S. ruralis*, and *Tortula atrovirens* (Sm.) Lindb.*, Tortula inermis* (Brid.) Mont. (e.g., [28,56,57,58,59]), which is expected. However, the approaches to desiccation phenomena in various moss species differ, making comparisons inappropriate and requiring novel comparative investigations for meaningful and result-supported inferences. Additionally, resilience to various stresses in distinct species can be inconsistent due to different sexes, ploidy and/or endopolyploidy, the presence of endophytes, crypto-speciation, biotic interactions, genotypes and/or ecotypes, chemical constituents, the season of moss collection, and many other factors, which can affect the results obtained both synergistically or antagonistically (e.g., [60,61,62,63]. Therefore, studies in controlled conditions and on clean moss material as well as comparative investigations are both welcomed and needed (e.g., [64,65]).

## 4. Materials and Methods

### 4.1. Collection of Samples

The specimens were randomly collected from dry siliceous rocks (leg./det. Marko Sabovljević) at Vysoké Tatry in the district of Poprad located in Northeastern Slovakia on 21 July 2022, in an anabiotic state and in an area of 100 m^2^. Seven equal patches of similar sizes consisting of pooled shoots were sampled and mixed for further experimentation to avoid individual peculiarities. On the collection day, the weather was fairly warm and dry before noon with a humidity of approx. 30%. This region experiences summer during July, the temperature is high 17°/low 12° (°C), with up to 10 rainy days on average.

#### Experimental Design

**Figure 3:**
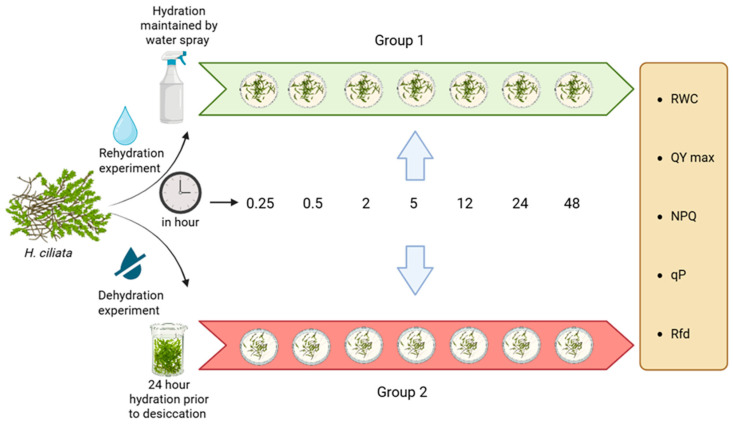
Experimental design: Response of *H. ciliata* towards dehydration and rehydration. The experiment was divided into 2 groups. Group 1 (rehydration experiment) and Group 2 (dehydration experiment). The Group 1 samples were kept on wet moist paper to rehydrate, and the moisture was maintained by regular water spraying of the paper substrate with 50 mL of water, twice a day in the morning and in the evening, while the Group 2 samples were fully hydrated for 24 h then allowed to air dry at room temperature for dehydration. The measurements were taken at multiple time points (0.25, 0.5, 2, 5, 12, 24, and 48 h). At each time point, the physiological parameters [RWC—relative water content, QY max—maximum quantum yield, NPQ—non-photochemical quenching, qP—photochemical quenching, and Rfd—fluorescence decline ratio] were measured in five replicates.

The dried mosses were separated into two main lines. Gametophore tips of 2 cm (20 per treatment) were subjected to hydration/dehydration for different time periods (Figure 3) and then the RWC and photosynthetic parameters were measured in time sequences (namely, 0.25 h, 0.5 h, 2 h, 5 h, 12 h, 24 h, and 48 h). The first group of dried mosses was transferred to moistened paper sheets to ensure gradual water uptake until the time of measurement, while the second group was allowed to dry at room temperature after 24 h of hydration. The two groups were then further divided into 7 subgroups to measure any changes in photosynthetic efficiency during 48 h of hydration/dehydration. At the end of the treatments, relative water content as well as photosynthetic efficiency were measured for each subgroup in five replicates. Each treatment was performed at a constant room temperature of 22 ± 2 °C, at a room air humidity of 70%, and light conditions of 100 μmol m^−2^ s^−1^.

### 4.2. Relative Water Content

For the RWC assay, 300 mg of moss tissue was taken from each subgroup. For each subgroup, the turgid weight (TW) of the moss plants was recorded after hydrating them for 24 h. The fresh weight (FW) was recorded at each designated time point of treatment (0.25 h, 0.5 h, 2 h, 5 h, 12 h, 24 h, and 48 h) within the subgroups of both of the tested lines. The same moss samples were used throughout. The dry weight (DW) was obtained by placing the moss plants in an oven at 80 °C for 48 h until they reached a constant weight. Each measurement was performed in five replicates.

Relative water content (RWC) was determined by the following formula [66]:

RWC (%) = [(FW − DW)/(TW − DW)] × 100

where FW is the fresh weight of the moss plants at each time point, as mentioned above, DW is the dry weight of the moss plants after oven drying to a constant weight, and TW is the fully hydrated weight of the moss plants. The value of the TW was reached by full submersion.

### 4.3. Chlorophyll Fluorescence

Moss plants in the experiment were subjected to chlorophyll fluorescence measurements along with RWC. A FluorCam 800 MF instrument (Photon Systems Instruments Ltd., Brno, Czech Republic) was used to determine the photosynthetic parameters for each of the subgroups. These measurements were performed in both experiment lines on dark-adapted samples (10 min in full darkness) using a saturating flash of light (2000 μmol m^−2^ s^−1^) for 1 s. Each measurement was performed in five replicates. The following parameters were measured:

(A) Maximum quantum yield of photosystem II (QY max.) = Fv/Fm (where Fv = Fm − F0, F0 represents the ground fluorescence in the dark-adapted state and Fm represents the maximum fluorescence during a saturating radiation pulse in the dark-adapted state).
(B) Photochemical quenching (qP).
(C) Non-photochemical quenching (NPQ).
(D) Fluorescence decline ratio (Rfd) = Fp/Ft (where Fp represents the fluorescence peak and Ft represents the steady-state fluorescence in the dark-adapted state).

The FluoCam was set to automatically calculate the values of qP and NPQ, as explained by Mucrchie and Lawson [67].

### 4.4. Statistical Analysis

The complete statistical analysis was performed using R software (v. 4.3.2) (R Core Team, 2023) [68]. Preliminary data exploration included the Shapiro–Wilk test for normality and Levene’s test for homogeneity of variances. Since the assumptions of the parametric tests were not met, nonparametric statistics were applied. Differences among treatment groups were analyzed using the Kruskal–Wallis test, and pairwise comparisons were performed using Dunn’s post hoc test with Benjamini–Hochberg *p*-value adjustments.

## 5. Conclusions

This study offers insights into the physiological resilience of *H. ciliata* under early desiccation–rehydration cycles, emphasizing its adaptation to desiccation. It exhibits a high capacity for rapid water uptake during short hydration periods, along with sustained relative water content (RWC) over extended periods. This trait seems to be facilitated by its mat-forming architecture and hyaline basal cells, enhancing its capillary water retention and minimizing water loss. The gradual decline in RWC during dehydration, alongside the preservation of photosynthetic efficiency variables, suggests that *H. ciliata* implements constitutive protective mechanisms to maintain photosystem II (PSII) integrity. The low NPQ values during desiccation imply limited photoinhibition and effective photoprotective regulation, while the high Rfd values during rehydration reflect the efficient recovery of photosynthetic performance. The observed delays in physiological shutdown during desiccation, as well as the uninterrupted metabolic activity in the early dehydration stages, indicate an anticipatory acclimation to impending anabiosis. These characteristics collectively support a desiccation-tolerant strategy characterized by both structural and physiological adaptations. Therefore, *H. ciliata* demonstrates a robust capacity for coping with abiotic stress, such as desiccation, and may serve as a potential model species reflecting functional adaptation to unpredictable environments.

## Data Availability

The original contributions presented in this study are included in the article. Further inquiries can be directed to the corresponding author.
